# The Role of Structural Representation in the Performance of a Deep Neural Network for X-ray Spectroscopy

**DOI:** 10.3390/molecules25112715

**Published:** 2020-06-11

**Authors:** Marwah M.M. Madkhali, Conor D. Rankine, Thomas J. Penfold

**Affiliations:** 1 Chemistry—School of Natural and Environmental Sciences, Newcastle University, Newcastle-Upon-Tyne NE1 7RU, UK; m.m.m.madkhali2@newcastle.ac.uk (M.M.M.M.); conor.rankine@newcastle.ac.uk (C.D.R.); 2 Department of Chemistry, College of Science, Jazan University, Jazan 45142, Saudi Arabia

**Keywords:** machine learning, deep neural network, Coulomb matrix, radial distribution curve, X-ray absorption spectroscopy, XANES, K-edge

## Abstract

An important consideration when developing a deep neural network (DNN) for the prediction of molecular properties is the representation of the chemical space. Herein we explore the effect of the representation on the performance of our DNN engineered to predict Fe K-edge X-ray absorption near-edge structure (XANES) spectra, and address the question: How important is the choice of representation for the local environment around an arbitrary Fe absorption site? Using two popular representations of chemical space—the Coulomb matrix (CM) and pair-distribution/radial distribution curve (RDC)—we investigate the effect that the choice of representation has on the performance of our DNN. While CM and RDC featurisation are demonstrably robust descriptors, it is possible to obtain a smaller mean squared error (MSE) between the target and estimated XANES spectra when using RDC featurisation, and converge to this state a) faster and b) using fewer data samples. This is advantageous for future extension of our DNN to other X-ray absorption edges, and for reoptimisation of our DNN to reproduce results from higher levels of theory. In the latter case, dataset sizes will be limited more strongly by the resource-intensive nature of the underlying theoretical calculations.

## 1. Introduction

Structural techniques, such as X-ray diffraction (XRD) and spectroscopy (XS), have made it possible to determine directly the structures of molecules and condensed matter systems and have had a huge influence across physics, chemistry, and biology. The proliferation of high-brilliance light sources such as 3^*rd*^ generation synchrotrons and X-ray free-electron lasers (XFELs) is helping to increase this influence of these techniques by facilitating the measurement of increasingly challenging systems such as operating catalysts [1,2] and short-lived reaction intermediates [3,4].

Besides providing element- and site-specific information on geometric structure, X-ray absorption spectroscopy (XAS) is also able to provide direct information on the electronic structure around the absorption site [5,6]. Indeed, XAS spectra are characterised by X-ray absorption edges that correspond to the excitation of core electrons to the ionisation threshold. The electrons are initially excited to unoccupied or partially-occupied orbitals at energies just below the ionisation potential (IP); these bound transitions, which form the pre-edge spectral features, provide detailed information about the nature of the unoccupied valence orbitals. At energies above the IP, resonances occur due to interference of the electron wave originating from the absorbing site with the electron wave scattered back from the neighbouring atoms. When the kinetic energy of the electrons is large, the scattering cross-section of the electrons is small, and information on the short-range geometric structure around the absorption site is accessible. This information is encoded in the extended X-ray absorption fine structure (EXAFS) region of the XAS spectrum, and is typically found >50 eV above the X-ray absorption edge. EXAFS encodes information about coordination numbers and the distance of nearest neighbours to the absorption site. In contrast, the X-ray absorption near-edge structure (XANES) region of the XAS spectrum, found at lower photoelectron energies (<50 eV above the X-ray absorption edge), is dominated by the interference of scattering pathways between multiple atoms. The XANES region encodes detailed information about coordination numbers, bond distances, and bond angles.

Extracting this information via analysis of the XAS spectrum demands detailed theoretical treatments [7,8]. The requisite theory for the analysis of EXAFS spectra is better developed than that required for the analysis of XANES spectra; the reason for this is that the higher-energy photoelectron is largely insensitive to the details and response of the potential [9,10]. The analysis of XANES spectra is more challenging and, despite significant progress [11,12,13], quantitative interpretation still necessitates resource-intensive theoretical treatments that can take hours/days to yield a single spectrum (cf. a few minutes for the calculation of an EXAFS spectrum). While the computational cost of such theoretical treatments is not prohibitive for a few calculations, it becomes so for the most challenging systems, e.g., disordered/amorphous systems, such as the surface of an operando catalyst [14,15,16], or dynamical processes [17,18,19,20,21]. A huge number of different and dynamically-evolving absorption sites must be modelled for a quantitative analysis, but to treat theoretically every possible chemically-inequivalent absorption site (or even to sample a meaningful number of such sites) is computationally challenging, resource-intensive, and time-consuming. This is a considerable challenge in the EXAFS analyses of such systems, which routinely necessitate molecular dynamics (MD) simulations with nuclear ensemble approaches [22] and, at present, an insurmountable one in many XANES analyses. Such analyses are out of reach for the majority of XAS users and, for the most complex systems, even expert theoreticians.

To address this, a number of recent works have explored supervised machine learning/deep learning algorithms with a view towards mapping the relationship between XANES spectra and the underlying electronic and geometric structure of materials [23,24,25,26,27,28,29,30,31]. In the majority of these recent works, the authors have focused on mapping the spectrum onto a property or structure, and these works have generally been system-specific or restricted to a narrow class of systems. Indeed, generally-applicable machine learning models capable of predicting XANES spectra for arbitrary absorption sites (i.e., machine learning models capable of learning ‘reverse’ mappings of the structure onto the spectrum) are lacking. We have addressed this by introducing a deep neural network (DNN) for the instantaneous prediction of Fe K-edge XANES spectra in a recent publication [32]. Our motivation is to accelerate the XANES analysis of complicated, disordered, and dynamically-evolving systems and, in these cases, it is unlikely that deriving a single structure from a XANES spectrum would prove useful or physically meaningful. In such cases, an accurate, high-throughput DNN for XANES simulation has great potential for revealing structure-spectrum relationships. Our DNN, which requires no input beyond the local geometric structure around an arbitrary Fe absorption site, is able to predict Fe K-edge XANES spectra with a mean squared error (MSE) below ca. 3% (evaluated on ca. 2000 unseen XANES spectra) after training with ca. 7000 theoretical examples [32]. We have demonstrated sub-eV accuracy on spectral peak positions and accuracy on peak positions that is an order of magnitude smaller than the spectral variations that our model was engineered to reproduce [32].

An important consideration when engineering a DNN for this task is the representation, or ‘featurisation’, of the input data: How should the local environment—the ‘chemical space’—around an arbitrary Fe absorption site be best represented to maximise the performance of the DNN? In this contribution, we explore the effect of representation on the performance of our DNN [32]. Using the Coulomb matrix (CM) [33,34,35] and pair-distribution/radial distribution curve (RDC) [36,37,38,39], we demonstrate that the latter not only leads to smaller mean squared error (MSE) but also achieves this faster and with smaller training set sizes, which greatly supports the development of a DNN which is generalisable across the whole periodic table.

## 2. Theory and Computational Details

### 2.1. Deep Neural Network

**Figure 1:**
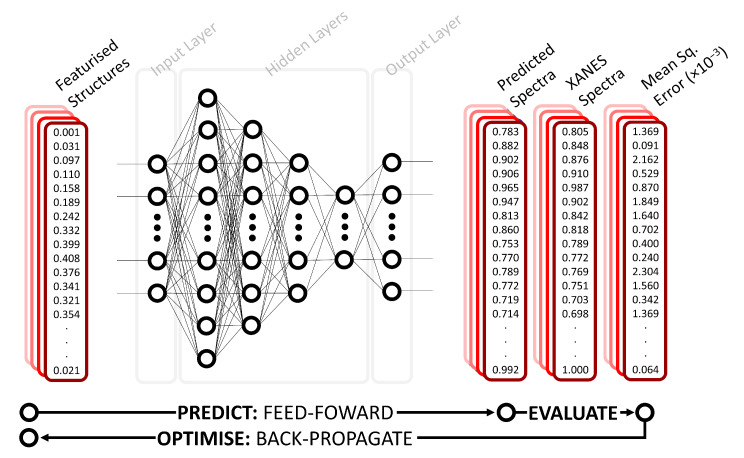
Schematic representation of the deep neural network (DNN) used in this work. The DNN takes the local environment around an atomic absorption site (featurised using either Coulomb matrix (CM) or radial distribution curve (RDC)) as input. This is passed through the network which consists of four hidden layers to output a predicted spectrum and mean squared error between the theoretical and predicted XANES spectra.

A schematic of our DNN is shown in Figure 1. Our DNN is based on the multilayer perceptron model (MLP); an MLP is a class of feed-forward neural network comprising an input layer, *n* hidden layers, and an output layer. The dimensions of the input layer in our DNN are determined by the representation used (see the “Representation” section). The first hidden layer comprises 1200 neurons and every subsequent layer is reduced in size by 30% relative to the preceding hidden layer; our DNN uses four hidden layers. The output layer comprises X neurons, defined by the discretization of our target XANES spectra.

The layers are all fully connected, or ‘dense’, i.e., each neuron in an arbitrary layer, *k*, is connected to every neuron in the preceding layer, k−1, via a matrix of weights, $w_{i,j}^{\left(k\right)}$. The pre-activation value of an arbitrary neuron in an arbitrary layer, $z_{k,j}$, is given by the linear combination of the input activations, $x_{\left(k-1\right)}$ (these being the output activations of each neuron in the preceding layer), and their respective weights: $z_{k,j}=\sum_{i}w_{ij}^{\left(k\right)}x_{(k-1),i}$. A non-linear activation function, g(z), is then applied to the pre-activation values to compute output activations for the neuron in the layer, ${\hat{y}}_{k,j}$; our DNN uses a hyperbolic tangent (tanh) activation function which constrains the possible values of $\hat{y}$ between −1 and +1. The resulting activations, obtained for an arbitrary neuron in an arbitrary layer as ${\hat{y}}_{k,j}=g\left(z_{k,j}\right)$, then serve as the input activations for the next layer, unless subject to an intermediate transformation. Information propagates through the MLP via a ‘feed-forward’ process until it arrives at the output layer. The activations of the output layer are then compared against target activations via evaluation of a cost function, $J\left(W\right)=\frac{1}{n}\sum_{i}^{n}\ell \left\{f\left(x^{\left(i\right)},W\right),y^{\left(i\right)}\right\}$, that quantifies the difference between the obtained, $f(x^{\left(i\right)},W)$, and expected, $y^{\left(i\right)}$, activations over a dataset of n samples as a function of the weights, W, and input activations, $x^{\left(i\right)}$. Our DNN uses a mean-squared error (MSE) cost function of the general form $J\left(W\right)=\frac{1}{n}\sum_{i}^{n}\left\{y^{\left(i\right)}-f\left(x^{\left(i\right)},W\right)\right\}$.

The derivatives of J(W) with respect to the internal weights, $\frac{\delta J(W)}{\delta W}$, can be calculated cost-effectively and used to adjust the internal weights such that J(W) is minimised; succinctly, the objective is to find a set of internal weights, $W^{*}$, for which $W^{*}=\underset{W}{\mathrm{argmin}}J\left(W\right)$.

Our DNN optimizes c.a. three million internal weights via sequential feed-forward and back propagation cycles. Gradients of the MSE cost function with respect to the internal weights are updated iteratively according to the Adaptive Moment Estimation (ADAM) algorithm. Gradients are estimated over minibatches of 100 samples. The learning rate for the ADAM algorithm, η, is set to $3\times 10^{-4}$.

The performance of our DNN is assessed via K-fold cross validation [40]. The data are randomly partitioned into *K* folds with K−1 folds kept in-sample to train the DNN and the remaining fold left out-of-sample to evaluate the performance of the DNN on unseen data. *K* evaluations are made such that every data sample appears in the out-of-sample testing set once, and in the in-sample training set K−1 times. The entire procedure can be repeated any number of times with different random *K*-fold partitions. The repeated evaluations of performance can be used to estimate an error. We mitigate the risk of overfitting our DNN to the training set by assessing the performance of each *K*-fold on the out-of-sample data only. We use five-fold cross-validation, i.e., an 80:20 in-sample/out-of-sample split, with five repetitions.

Our DNN also utilises dropout [41]; dropout is a regularisation technique that sets the activations of a certain fraction of the neurons in each layer are set to zero during the feed-forward/back-propagation procedure. Utilising dropout encourages a DNN to distribute weights probabilistically, and works to mitigate layers adapting to correct for mistakes in other layers; the latter behaviour would otherwise lead to overfitting, as these adaptions will not generalise well beyond the in-sample dataset. Our DNN uses a dropout of 15%.

Our DNN is programmed in Python 3 with the TensorFlow/Keras [42,43] API. All hyperparameters were determined via Bayesian optimisation using the GPyOpt [44,45] module, as in Reference [32].

### 2.2. Representation

We trial two alternative representations of chemical space: The CM and RDC.

The CM representation ($M_{IJ}$) [33,34,35] is constructed as:

(1) $$M_{IJ}=\left\{\begin{array}{c} \frac{1}{2}Z_{I}^{2.4}\;\;\forall I=J\\ \frac{Z_{I}Z_{J}}{|R_{I}-R_{J}|}\;\forall I\neq J \end{array}\right.$$

*Z* is the nuclear charge of an atom and R is the position of the atom in Cartesian space. $M_{IJ}$ is a symmetric matrix of dimensions N×N where *N* is the upper limit on the number of atoms designated for inclusion in the CM. The off-diagonal elements of $M_{IJ}$ correspond to a Coulombic repulsion term between atoms *I* and *J* and the on-diagonal elements of $M_{IJ}$ correspond to the Coulomb potentials of the free atoms. The rows of $M_{IJ}$ are sorted in descending order according to their Euclidean ($L^{2}$) norms, $\left|\right|M_{I}\left|\right|$, i.e., a permutation of the rows and columns is found that satisfies the inequality:

(2) $$\left|\right|M_{I}\left|\right|\geq \left|\right|M_{I+1}\left|\right|\;\forall I$$

The upper triangle of $M_{IJ}$ is then taken and flattened row-wise to yield a feature vector of length $\frac{1}{2}\left(N^{2}+N\right)$. Practicably, this feature vector should have the same length, regardless of the size of the system it encodes, if it is to be input into a neural network. If the system contains more than *N* atoms, the closest *N* atoms to the absorbing atom are used to construct the CM and the rest are discarded; if the system contains less than *N* atoms, the remaining rows and columns of the CM are zero-filled. The sorted CM representation is unique, invariant with respect to atomic indexing, translations, and rotations of the chemical space that it describes, and its construction requires no explicit information on chemical bonding [33,34,35].

Where CM featurisation is used in this work, N=20.

The RDC representation [36,37,38,39] encodes local chemical space as an intensity distribution ($f_{RDC}$) as a function of equally-distributed values of *R*, where the intensity is defined:

(3) $$f_{RDC}=\sum_{I}^{n}\sum_{J>I}^{n}Z_{I}Z_{J}exp^{-\alpha {\left(r_{IJ}-R\right)}^{2}}$$

**Figure 2:**
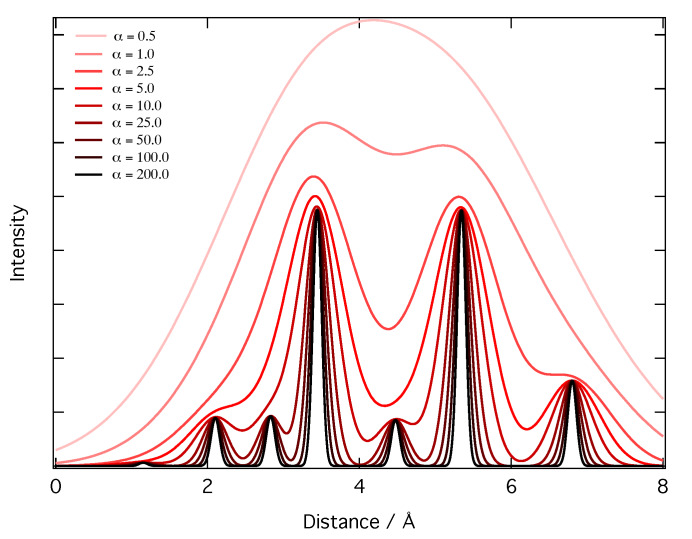
An RDC for an arbitrary system; the intensity (the probability of finding an interatomic distance, $r_{IJ}$ at some arbitrary distance, *r*) is plotted as a function of the distance, *r*, for nine values of α between 0.5 and 200.0. A larger value of α increases the decay of the exponential at each value of *r*, increasing the resolution of the RDC; very large values of α yield sparse RDCs.

$Z_{I}$ and $Z_{J}$ are the nuclear charges of atoms *I* and *J*, respectively. $r_{IJ}$ is the distance between atoms *I* and *J*, and R is a vector obtained by discretizing a linear interpolation between zero and twice the cutoff radius around the absorption site (defining the maximum pairwise distance that can be encoded by the RDC). α is a smoothing parameter that controls the resolution of the RDC. As α is increased, so too is the detail that is visible in RDC, but if α is too large, the RDC starts to become sparse. This is illustrated in Figure 2.

Like the CM representation, the RDC representation is invariant with respect to atomic indexing, translations, and rotations of the chemical space that it describes, and its construction requires no explicit information on chemical bonding [39]. It does not have to be weighted by $Z_{I}$ and $Z_{J}$ alone; indeed, it is possible to construct property-weighted RDCs using any relevant atomic property (e.g., electron affinity, electronegativity, van der Waals radius, ect.) [46,47] to engineer the descriptor for a specific purpose.

An additional advantage of the RDC is that $f_{RDC}$ can be discretised to yield a feature vector of constant length [39,46,47] regardless of the size of the chemical space it describes, i.e., CM featurisation encodes information on a fixed number of atoms and, consequently, a fixed number of interatomic distances, while RDC featurisation flexibly encodes information on all atoms and interatomic distances below a cutoff radius as per specification of R.

Where RDC featurisation is used in this work, α=10.0 and $R=0.0\overset{1.2}{⇁}800.0$ pm.

The CM and RDC representations are useful descriptors on account of their simplicity; both require very little space in memory, and the requisite operations for their construction are easily vectorisable; large datasets can be featurised quickly, and can typically fit in the memory in their entirety.

### 2.3. Dataset

Our dataset comprises 9040 unique Fe-containing structures harvested from the Materials Project Library via the Materials Project API. Fe K-edge XANES spectra for one arbitrary absorption site per structure have been calculated using multiple scattering theory as implemented in the FDMNES package [12]. The Fe K-edge XANES calculations employed a self-consistent muffin-tin-type potential of radius 6.0 Å around the absorbing site. The interaction with the X-ray field was described using the electric quadrupole approximation, and scalar relativistic effects were included. To transform the computed cross-sections into XANES spectra that can be compared to experiment, the cross-sections need to be convoluted with a function that accounts for the core-hole-lifetime broadening, instrument response, and many-body effects, e.g., inelastic losses. Throughout this work, this convolution has been performed using an energy-dependent arctangent function via an empirical model close to the Seah-Dench formalism [48]:

(4) $$\Gamma =\Gamma_{i}+\Gamma_{f}\left(\frac{1}{2}+\frac{1}{\pi }\arctan\left(\frac{\pi }{3}\frac{\Gamma_{f}}{E_{w}}\left(\frac{E-E_{f}}{E_{c}}-\frac{E_{c}^{2}}{{\left(E-E_{f}\right)}^{2}}\right)\right)\right)$$

Γ is defined over the energy scale, E, of the XANES spectrum as per specification of the core-level and final-state widths ($\Gamma_{i}$ and $\Gamma_{f}$, respectively), and the centre and width of the arctangent function ($E_{c}$ and $E_{w}$, respectively). The arctangent convolution is performed as implemented in the FDMNES package [12].

The arctangent convolution is only applied as a post-processing step on XANES spectra estimated by our DNN; our dataset comprises only unconvoluted cross-sections, and our DNN learns from these unconvoluted cross-sections.

## 3. Results

### 3.1. Performance of the Deep Neural Network

**Figure 3:**
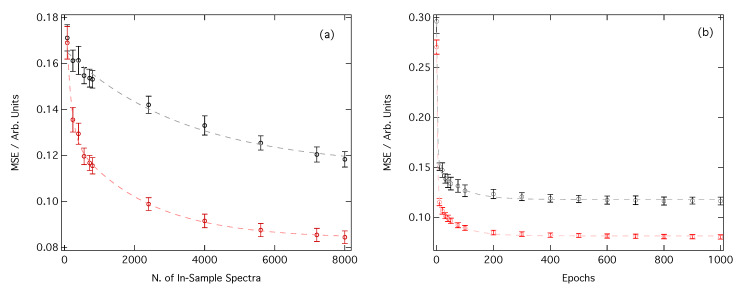
(**a**) Evolution of the mean squared error (MSE) as a function of the number of in-sample spectra accessible to the DNN. (**b**) Evolution of the MSE as a function of the number of forward passes through our dataset (‘epochs’). The local environment around each Fe absorption site has been featurised either as a CM (black) or RDC (red). Data points are averaged over 100 K-fold cross-validated evaluations; error bars indicate one standard deviation.

Figure 3a shows the MSE as a function of the number of in-sample spectra accessible to our DNN during the learning process. The local environment around each Fe absorption site has been featurised either as a CM (black) or RDC (red). In the small-sample limit (ca. 100 in-sample spectra), both representations exhibit similar performance with the MSE of 0.17. However, as the number of in-sample spectra accessible to our DNN is increased, an almost linear improvement in MSE is seen when CM featurisation is used and a MSE of 0.12 is obtained for the large-sample limit (ca. 9000 in-sample spectra). In contrast, RDC featurisation gives a rapid initial improvement, delivering a much smaller MSE than can be achieved via CM featurisation. Beyond ca. 2000 in-sample spectra, the improvement in the MSE begins to slow, but the final MSE in the large-sample limit (ca. 0.08) is still significantly lower than the MSE that can be achieved using CM featurisation.

Figure 3b illustrates the performance during the training of the DNN, shown as a function of the number of forward passes through our dataset. While, as seen in Figure 3a, we achieve a lower MSE for the RDC representation, in both cases it is observed that the DNN can be optimised in <500 forward passes through the dataset. This is achievable in as little as five to ten minutes if graphical processing unit (GPU) acceleration is used.

### 3.2. Predictions of Peak Position and Intensity

**Figure 4:**
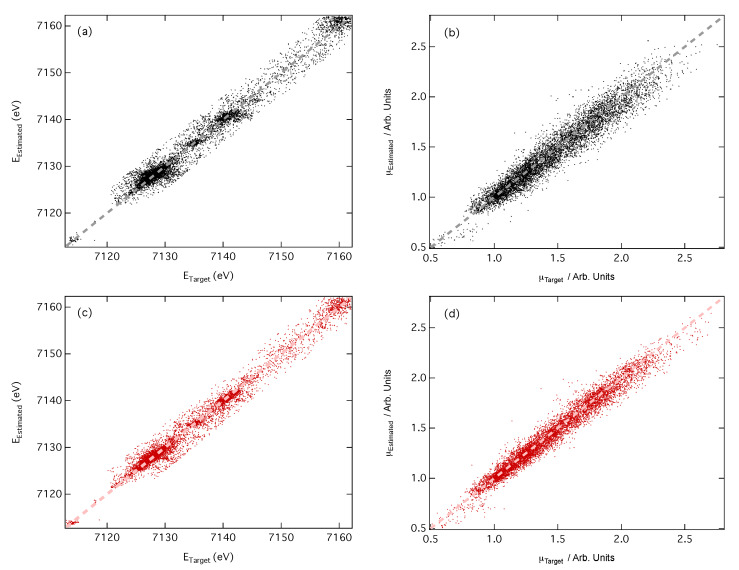
Parity plots of estimated and target peak positions on the (**a**,**c**) energy (*E*_Target_ and *E*_Estm._, respectively) and (**b**,**d**) intensity (μ_Target_ and μ_Estm._, respectively) scales; (**a**,**b**) use the CM representation, while (**c**,**d**) use the RDC representation.

When predicting XANES spectra, accurate reproduction of the positions and intensities of above-ionisation resonances is crucial, as these directly encode the structural information in the spectrum. Figure 4 shows parity plots of the difference between the estimated and target peak positions on the energy (*E*_Target_ and *E*_Est._) and intensity (μ_Target_ and μ_Est._) scales. The upper (Figure 4a,b) and lower panels (Figure 4c,d) display the results from CM and RDC featurisation, respectively.

In both cases, a strong linear relationships are evidenced by the coefficients of determination, $R^{2}$, which are 0.974 and 0.930 for energy and intensity, respectively, if CM featurisation is used, and 0.986 and 0.973 for energy and intensity, respectively, if RDC featurisation is used. As expected from the training curve shown in Figure 3a, the RDC representation performs slightly better exhibiting a lower $R^{2}$ in both cases as expected from the narrower spread which is visible in Figure 4.

### 3.3. Predictions of Spectra

**Figure 5:**
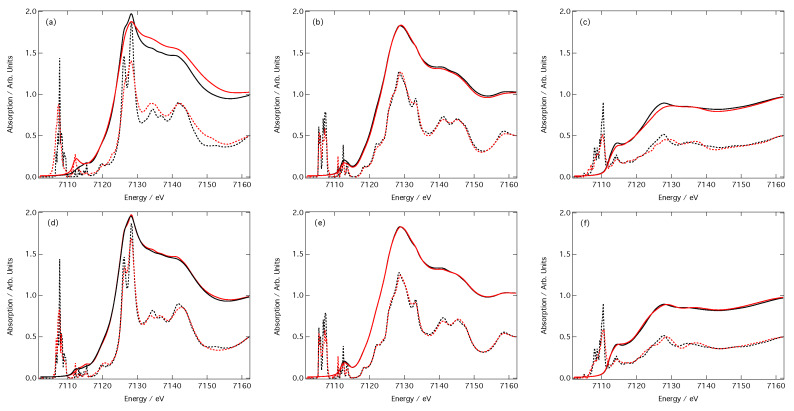
Arctangent-convoluted (solid) and unconvoluted (dashed) target (black) and out-of-sample DNN-estimated (red) Fe K-edge X-ray absorption near-edge structure (XANES) spectra for absorption sites in (**a**,**d**) $C_{6}{\mathrm{Al}}_{2}{\mathrm{Fe}}_{4}O_{15}$, (**b**,**e**) FeOF, and (**c**,**f**) $Sm_{2}Fe_{17}H_{3}$. Spectra belong to the first centile when performance is ranked over all out-of-sample DNN estimations by MSE. Spectra in panels a–c and d–f were obtained using the CM and RDC representations, respectively. Amplitudes of all unconvoluted spectra have been reduced by half for clarity.

Figure 5 compares six computed XANES spectra with their corresponding out-of-sample DNN estimations. The dashed lines represent the computed and predicted cross-sections (scaled by 50% for clarity) and the solid lines represent the computed and predicted XANES spectra post-convolution of the cross-sections with the arctangent function (Equation (4)). These XANES spectra all belong to the first centile when performance is ranked over all out-of-sample DNN estimations by MSE.

The top three XANES spectra (Figure 5a–c) were obtained using CM featurisation, while the bottom three (Figure 5d–f) were obtained using RDC featurisation. In the latter case, the DNN- estimated XANES spectra can hardly be distinguished from the target XANES spectra; while discrepancies can be observed in the unconvoluted cross-sections on which our DNN is trained, the differences are negligibly small and can be considered insignificant once the arctangent convolution has been applied. In contrast, differences between the DNN-estimated and target XANES spectra are amplified when using CM featurisation. Inspection of the unconvoluted cross-sections suggests that these differences have their origin in the estimated intensities of peaks; this is most apparent in Figure 5a. CM featurisation performs less effectively than RDC featurisation on estimated peak intensities, as also evidenced in Figure 4.

**Figure 6:**
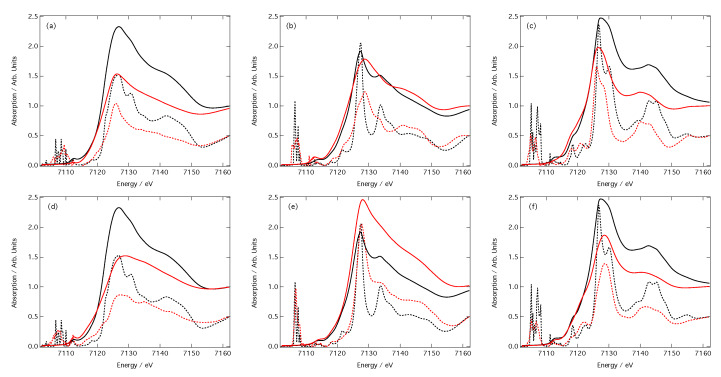
Arctangent-convoluted (solid) and unconvoluted (dashed) target (black) and out-of-sample DNN-estimated (red) Fe K-edge XANES spectra for absorption sites in (**a**,**d**) $Fe_{3}H_{36}C_{12}S_{6}{\left(\mathrm{Br}O_{2}\right)}_{3}$, (**b**,**e**) $\mathrm{KF}e_{2}F_{6}$, and (**c**,**f**) $Li_{7}Fe_{3}O_{10}$. Spectra belong to the ninety-nineth centile when performance is ranked over all out-of-sample DNN estimations by MSE. Spectra in panels a–c and d–f were obtained using the CM and RDC representations, respectively. Amplitudes of all unconvoluted spectra have been reduced by half for clarity.

Figure 6 shows the three samples spectra, optimised using the CM (panels a–c) and the RDC (panels d–f) representation, drawn from the ninety-nineth centile, i.e., the mostly poorly predicted spectra. In this case, as with previous work [32], the principle reason that these are in the lowest centile is due to their underestimation of the spectral intensity that compounds across the energy scale. This is especially true in the case of the RDC representation which finds peaks in the right position. This is less so for the CM representation, which is especially apparent in Figure 6b,c, for which larger deviations are observed.

## 4. Discussion and Conclusions

Appropriate featurisation is crucial for achieving best-in-class performance from a DNN. In this contribution, we have outlined the effect that the choice of featurisation (CM or RDC featurisation) has on the performance of our DNN [32] engineered for the prediction of XANES spectra at Fe K-edge. In both cases, a MSE of ≤0.12 is readily achievable in only a few minutes of real-time learning, and the example estimations of out-of-sample XANES spectra shown in Figure 5 and Figure 6 demonstrate that both representations are able to deliver qualitative predictions of out-of-sample XANES spectra, even in the ninety-nineth centile. However, Figure 3 demonstrates that convergence of the DNN during the learning process is faster, better if one is restricted to the small-sample limit, and ultimately achieves a lower final MSE in the large-sample limit. These results evidence that the RDC is to be preferred over the CM as a representation of chemical space for this particular problem and DNN architecture.

At this point, it is important to highlight an important difference between the CM and RDC representations. Throughout this work we have limited the dimensions of $M_{IJ}$ to 20×20. Optimisation of our DNN has lead us to identify N=20 as the optimal CM dimension, i.e., that which gives the lowest MSE as evaluated on out-of-sample examples, within the limit of the possible values of *N* that are large enough to capture the necessary structural information, but not so large as to increase the propensity for overfitting. The maximum radius around an absorption site encoded into a CM of dimensions 20×20 is consequently system-dependent, e.g., it depends on the identities of the neighbouring atoms around the absorption site and, by extension, the packing density of the system to be featurised.

**Figure 7:**
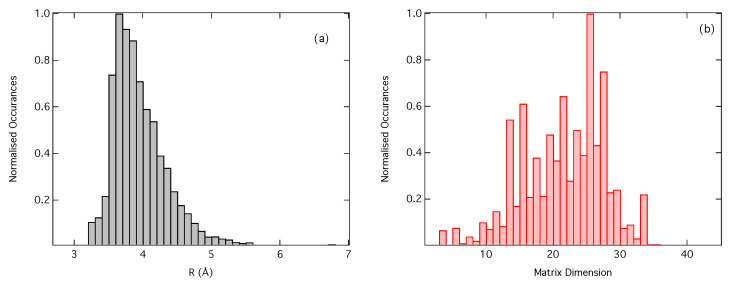
(**a**) Histograms of (**a**) the maximum radii around each Fe absorption site encoded in a CM of dimensions 20×20, and (**b**) the necessary CM dimension, *N*, required to encode a radius of 4.0 Å around each Fe absorption site in our dataset. Histograms are normalised.

Figure 7a shows a histogram of the maximum radius around the absorption site encoded into $M_{IJ}$ when the dimensions are limited to 20×20. The modal radius is ca. 3.5 Å, which is in close agreement with, albeit slightly smaller than, the optimal cutoff radius identified for RDC featurisation (4.0 Å) and this cutoff radius encodes approximately two coordination spheres around the absorption site. The smaller cutoff radius suggests that it is harder for CM featurisation to encode effectively all of the geometric information required to reproduce the XANES spectra as accurately as when using RDC featurisation. Figure 7b shows the reverse, i.e., a histogram of the CM dimensions when the radius around the absorbing atoms is set to 4.0 Å. Here the model dimension is around 25 × 25, meaning that the RDC featurisation, on average, can describe the effect of a larger number of atoms around the absorbing atom.

In summary, the performance of our DNN demonstrates that it is possible to develop a highly generalisable neural network for the prediction of XANES spectra at a specific absorption edge for an arbitrary absorption site, and that the RDC is a robust local descriptor for this purpose. This represents a highly encouraging starting point for our proof-of-principle demonstration which can be developed in a number of ways. Firstly, our theoretical XANES spectra (from which our DNN learns) are calculated under the muffin-tin approximation, and although this represents a computationally cost-effective choice for developing the underlying method, it is clear that the usefulness of our DNN can be considerably improved by moving beyond this. Secondly, the training set from which our DNN learns is composed of perfectly-ordered, homogeneous crystalline systems. While we have previously demonstrated [32] that it can be applied to situations outside of this scope, the sensitivity of our DNN to irregularities in the bulk such as vacancies, defects, undercoordinated sites, and the effects of lattice stress remains unclear. Finally, our DNN primarily considers only the local geometric environment around the absorption site of interest, and its ability to describe the changes in electronic charge state of the absorbing atom and therefore reproduce edge shifts is still uncertain. This could be incorporated as a post-processing step by simply shifting the predicted XANES spectra; this is commonly used for good approximation in time-resolved XAS experiments [49,50]. It remains desirable, however, to have this included from first principles. These aspects will be the focus of future work.
